# Platelet count, aspirin use, and characteristics of host inflammatory responses in colorectal cancer

**DOI:** 10.1186/s12967-019-1950-z

**Published:** 2019-06-13

**Authors:** Juha P. Väyrynen, Sara A. Väyrynen, Päivi Sirniö, Ilkka Minkkinen, Kai Klintrup, Toni Karhu, Jyrki Mäkelä, Karl-Heinz Herzig, Tuomo J. Karttunen, Anne Tuomisto, Markus J. Mäkinen

**Affiliations:** 1 Cancer and Translational Medicine Research Unit, University of Oulu, POB 5000, 90014 Oulu, Finland; 2 Department of Pathology, Oulu University Hospital and Medical Research Center Oulu, POB 21, 90029 Oulu, Finland; 3 Department of Oncologic Pathology, Dana-Farber Cancer Institute and Harvard Medical School, 450 Brookline Ave, Boston, MA 02215 USA; 4 Department of Medical Oncology, Dana-Farber Cancer Institute and Harvard Medical School, 450 Brookline Ave, Boston, MA 02215 USA; 5 Research Unit of Surgery, Anesthesia and Intensive Care, University of Oulu, POB 5000, 90014 Oulu, Finland; 6 Department of Surgery, Oulu University Hospital and Medical Research Center Oulu, POB 21, 90029 Oulu, Finland; 7 Research Unit of Biomedicine and Biocenter of Oulu, University of Oulu, POB 5000, 90014 Oulu, Finland; 8 Oulu University Hospital and Medical Research Center Oulu, POB 21, 90029 Oulu, Finland; 9 Department of Gastroenterology and Metabolism, Poznan University of Medical Sciences, ul. Szpitalna 27/33, 60-572 Poznan, Poland

**Keywords:** Thrombocytosis, Colorectal cancer, Inflammation, Prognosis, Aspirin, Platelet, CRP, Cytokine

## Abstract

**Background:**

Platelets not only contribute to hemostasis but also to the regulation of inflammatory reactions and cancer pathogenesis. We hypothesized that blood platelet count would be associated with systemic inflammation, the densities of tumor infiltrating immune cells, and survival in colorectal cancer (CRC), and these relationships could be altered by aspirin use.

**Methods:**

We measured blood platelet count in a cohort of 356 CRC patients and analyzed its relationships with tumor and patient characteristics including aspirin use, markers of systemic inflammation (modified Glasgow Prognostic Score, mGPS; serum levels of CRP, albumin, and 13 cytokines), blood hemoglobin levels, five types of tumor infiltrating immune cells (CD3, CD8, FoxP3, Neutrophil elastase, mast cell tryptase), and survival.

**Results:**

Platelet count inversely correlated with blood hemoglobin levels (p < 0.001) and positively correlated with serum levels of CRP and multiple cytokines including IL-1RA, IL-4, IL-6, IL-7, IL-8, IL-12, IFNγ, and PDGF-BB (p < 0.001 for all), while aspirin use was not associated with the levels of systemic inflammatory markers. High platelet count was also associated with high mGPS (p < 0.001) but did not show statistically significant multivariable adjusted associations with the densities of tumor infiltrating immune cells. Higher platelet counts were observed in higher tumor stage (p < 0.001), but platelet count or aspirin use were not associated with patient survival.

**Conclusions:**

High platelet count is associated with systemic inflammation in CRC. This study could not demonstrate statistically significant associations between platelet count, aspirin use, and the densities of tumor infiltrating immune cells.

**Electronic supplementary material:**

The online version of this article (10.1186/s12967-019-1950-z) contains supplementary material, which is available to authorized users.

## Background

Colorectal cancer (CRC) is one of the most common malignancies and causes of cancer death [1]. The most important prognostic factor is tumor stage [2, 3]. However, each tumor and patient is unique [4], and more exact categorization based on the features of the tumor and the host could improve our understanding on CRC and enable more individualized disease classifications and treatments.

Platelets are anucleate cell fragments generated by megakaryocytes in the bone marrow and also in other organs including the lung [5, 6]. Their primary function is to contribute to hemostasis and prevent bleeding [5, 7]. However, they are also involved in multiple other physiological processes, including inflammation, immunity, angiogenesis, and vessel remodeling [7]. Thrombocytosis, i.e., increased blood platelet count, is either reactive (secondary thrombocytosis) or caused by a clonal bone marrow (myeloproliferative) disorder [8]. The drivers of secondary thrombocytosis include acute or chronic infection or inflammation, iron deficiency, hemolytic anemia, asplenia, and cancer [8].

A multitude of evidence links platelets with cancer pathogenesis [5, 7]. For example, platelets, reacting to the modified tumor vasculature, can release growth factors or enzymes that stimulate cancer cell proliferation or angiogenesis or degrade extracellular matrix [7]. Additionally, platelets and their released mediators such as cytokines can contribute to the regulation of tumor associated inflammatory reactions [7]. Thrombocytosis is common in CRC, and three recent meta-analyses, based on 30 studies [9], 16 studies [10], and 9 studies [11] indicate that elevated preoperative platelet count is associated with shorter overall and disease-free survival in CRC. However, comparative analyses including additional prognostic parameters such as lymphatic invasion would be required to establish elevated platelet count as a relevant prognostic indicator in CRC.

Aspirin is one of the most commonly used drugs, with role as analgesic, antipyretic, and agent for cardiovascular prophylaxis [12]. The cardioprotective effects of aspirin are thought to be primarily based on inhibition of platelet production of TXA2 (thromboxane A2) [13], resulting in reduced platelet activation and aggregation. Substantial evidence from observational studies and randomized controlled trials support the efficacy of aspirin in the prevention of cancer, especially CRC [12, 14, 15]. Moreover, aspirin use has been associated with improved outcome in CRC [12, 16]. Earlier studies have indicated that the benefit of aspirin may be related to the tumor characteristics, such as PTGS2 expression [16] or PIK3CA mutation [17], but also platelet-related mechanisms of action have been suggested [12]. Thus, we hypothesized that the beneficial survival effect of aspirin in CRC could be stronger for patients with high platelet counts.

Colorectal cancer can elicit an anti-tumor immune response that restricts tumor growth and is associated with improved survival [18–20]. However, a systemic inflammatory response to cancer is associated with adverse outcome and may facilitate cancer progression by recruiting or mobilizing tumor-promoting inflammatory cells, attenuating the anti-tumor immune response, enhancing tumor cell migration and circulating tumor cell survival, and modifying the parenchyma of the pre-metastatic sites [21]. We have previously shown that CRC patients have increased serum levels of IL-6, IL-7, IL-8, and platelet-derived growth factor BB (PDGF-BB), and the patients with distant metastasis have higher serum levels of IL-1ra, IL-4, IL-6, IL-7, IL-8, MCP-1, and PDGF-BB [22]. Although platelets are considered important regulators of tumor associated inflammatory reactions [7, 21], the relationships between platelet count, serum cytokine milieu, and tumor infiltrating immune cells in CRC are currently poorly understood.

In this study, we analyzed blood platelet counts in a prospectively recruited cohort of 356 CRC patients and studied its relationships with patient characteristics including aspirin use; markers of systemic inflammation (modified Glasgow Prognostic Score, mGPS; serum levels of CRP, albumin, and 13 cytokines) and blood hemoglobin levels; tumor characteristics including five types of tumor infiltrating immune cells (CD3, CD8, FoxP3, Neutrophil elastase, mast cell tryptase); and survival.

## Methods

### Patients

This study is based on an earlier described prospectively recruited cohort of surgically treated stage I–IV CRC patients, operated in Oulu University Hospital in 2006–2014 (n = 356; Additional file 1: Table S1) [22–24]. All the patients were required to sign a written informed consent to participate. The study was approved by the Ethics Committee of Oulu University Hospital (58/2005, 184/2009) and was performed in accordance with the Declaration of Helsinki. The clinical details, including the list of patients’ medication, were collected from the clinical records and follow-up data from the clinical records and from Statistics Finland [25, 26]. Time to recurrence (TTR) was defined as time from the operation to the recurrence of the same cancer, cancer specific survival (CSS) was defined as time from the operation to death from the same cancer, and overall survival (OS) was defined as time from the operation to death, irrespective of cause. Preoperative staging of rectal cancer was conducted with magnetic resonance imaging, and most rectal cancer patients with cT3 or cT4 tumors (n = 69) received preoperative radiotherapy or chemoradiotherapy (RT/CRT). Additionally, one colon cancer patient received neoadjuvant treatment. The study was designed in accordance with the REMARK criteria [27].

### Blood analyses

Blood and serum samples were collected preoperatively [22]. Blood platelet count, blood Hb, erythrocyte MCV, serum CRP and serum albumin were measured in the laboratory of Oulu University Hospital [22, 28]. Anemia was defined according to WHO criteria as blood Hb levels < 120 g/L in women and < 130 g/L in men [23]. It was classified according to erythrocyte MCV levels as microcytic (MCV < 80 fL), normocytic (MCV 80–100 fL), and macrocytic (MCV > 100 fL) [23]. CRP and albumin were used to calculate modified Glasgow Prognostic Score (mGPS): mGPS0, serum CRP ≤ 10 mg/L and serum albumin ≥ 35 g/L or < 35 g/L; mGPS1, serum CRP > 10 mg/L and serum albumin ≥ 35 g/L; mGPS2, serum CRP > 10 mg/L and serum albumin < 35 g/L) [18, 29]. In patients operated between 2006 and January 2010 (n = 148), serum analysis of 27 cytokines was performed with Bio-Plex Pro Human 27-Plex Cytokine Panel (Bio-Rad, Hercules, CA, USA), as described earlier [22]. As described earlier in more detail, 14 cytokines had many values outside the assay detection limits, and 13 cytokines (IL-1ra, IL-4, IL-6, IL-7, IL-8, IL-9, IL-12, IFN-γ, CXL10, CCL2, CCL4, CCL11, and PDGF-BB) with less than four values outside the assay working range were included in this study [22, 30].

### Histopathological analysis

Histopathological analysis was conducted using the hematoxylin and eosin stained sections. Tumor stage was reclassified according to TNM8, and the grading was conducted according to the WHO criteria. Lymphatic invasion was defined as tumor cells present in vessels with an endothelial lining but lacking a muscular wall, while blood vessel invasion was defined as tumor cells in vessels with a thick muscular wall or in vessels containing red blood cells [31].

### Immunohistochemistry

Tissue microarrays were constructed for immunohistochemical analysis, consisting of 1–4 cores of 3.0 mm diameter for each tumor, depending on the size of the tumor, from the invasive margin (IM) and the tumor center (CT) [23, 32]. Immunohistochemistry for mismatch repair (MMR) enzymes MLH1, MSH2, MSH6, and PMS2 was conducted, as described earlier, to evaluate MMR enzyme status [23, 28, 33]. BRAF V600E specific VE1 immunohistochemistry was conducted with Ventana Bench-Mark XT immunostainer (Ventana Medical Systems, Tucson, AZ) [34], to evaluate *BRAF* mutation status. Our earlier study indicated that the method had a sensitivity of 100% and a specificity of 99.3% in detecting *BRAF* V600E mutation [34]. The densities of five types of tumor infiltrating immune cells at the invasive margin (IM) and center of the tumor (CT) were determined with an ImageJ based computer assisted analysis method [35], as described earlier [24, 36]. Intraepithelial (IEL) CD3^+^ and CD8^+^ lymphocytes were counted manually from the captured images, because the method could not segregate intraepithelial cells from those in tumor stroma [36]. The antibodies and protocols for immunohistochemistry are specified in Additional file 1: Table S2. All the histological and immunohistochemical analyses were performed blinded to other data.

### Statistical analyses

The statistical analyses were conducted using R version 3.5.1 (R Foundation for Statistical Computing, Vienna, Austria), using the packages tidyverse (v.1.2.1), survival (v.2.42-6), survminer (v.0.4.3), plotROC (v.2.2.1), car (v.3.0-2), lm.beta (v.1.5-1), and powerSurvEpi (v.0.1.0), or IBM SPSS Statistics for Windows, version 24.0 (IBM Corp., Armonk, NY). The statistical significances of the associations between categorical and continuous variables were analyzed by independent samples t-test or Mann–Whitney test (comparing two classes), or one-way analysis of variances (ANOVA) or Kruskal–Wallis test (comparing three or more classes), as appropriate, while the statistical significances of the associations between two categorical variables were analyzed with χ^2^ test. Pearson correlation coefficients (r) were used to determine the correlation between two continuous variables. To normalize their distribution, logarithmic transformation was applied to variables with positive skewness. Multiple linear regression models were used in multivariable analysis of the associations between blood platelet count, systemic inflammatory markers/tumor infiltrating immune cells, and selected clinicopathological factors. The normality of the residuals, homoscedasticity, and linearity assumptions were checked by a histogram and a normal probability plot of the residuals and by a scatterplot of residuals vs. predicted values. Cytoscape, an open source software platform for visualizing complex networks, was used in creating a 2D visualization of the relationships between blood platelet count, blood Hb levels, and serum levels of systemic inflammatory markers with the Prefuse force directed algorithm weighted by the statistical significances of the correlations between individual variables [37]. Receiver operating characteristics (ROC) analysis was used to evaluate the capacity of platelet count to discriminate the survivors from non-survivors. The survival outcomes of the patient subgroups were analyzed with Kaplan–Meier curves, log-rank test, and Cox proportional hazards regression analysis. In multivariable Cox models, the cases with missing data were assigned to the majority category of a given covariate to limit the degrees of freedom of the models. We confirmed that excluding cases with missing data on any of the covariates did not substantially alter the results (data not shown). An interaction between platelet count and aspirin use was assessed by including the product interaction term of these variables in the model. All p values are two-tailed. As our primary hypothesis testing, we evaluated the relationships between blood platelet count and TTR, CSS, and OS. For these primary, confirmatory survival analyses, we used α level of 0.05, while for the other exploratory analyses, we used α level of 0.005 [38], and regarded the results with p = 0.05–0.005 as of borderline statistical significance and interpreted them cautiously. Based on the most recent meta-analysis, we expected to observe a HR of 2.11 for the association between high platelet count and OS [11]. The statistical power (1−β) to detect this HR was estimated to be 0.98 at α level of 0.05, 0.92 at α level of 0.01, 0.88 at α level of 0.005, and 0.76 at α level of 0.001 (Additional file 1: Figure S1).

## Results

### Platelet count and clinicopathological features

Among the 356 CRC patients, mean platelet count was 294.9 × 10^9^/L (SD 93.4 × 10^9^/L) (Table 1). High platelet count, studied as a continuous variable, was associated with female gender (p = 0.002), proximal tumor location (p < 0.001), high TNM stage (p < 0.001), especially high T-class (p = 0.003) and M-class (p = 0.001), and normocytic and microcytic anemia (p < 0.001). High platelet count also showed tendency towards an association (borderline statistical significance considering multiple hypothesis testing) with young patient age (p = 0.044) and MMR deficiency (p = 0.006). Platelet count had no association with aspirin use (p = 0.396).

**Table 1:** Relationships between blood platelet count and clinicopathological characteristics

Variable	Blood platelet count (10^9^/L), mean (sd)	p value
All patients, n = 356	294.9 (93.4)	
Age		
< 65, n = 130	308.0 (94.0)	0.044
= 65, n = 226	287.4 (92.4)	
Gender		
Male, n = 190	280.9 (97.3)	0.002
Female, n = 166	310.9 (86.3)	
Body mass index		
< 25, n = 123	298.7 (102.2)	0.688
25–30, n = 146	288.8 (81.5)	
> 30, n = 78	293.5 (100.5)	
Tumor location		
Proximal colon, n = 123	319.4 (90.6)	< 0.001
Distal colon, n = 73	301.7 (102.2)	
Rectum, n = 160	273.0 (86.4)	
Neoadjuvant therapy in rectal cancer patients		
No, n = 91	279.0 (95.7)	0.295
Yes, n = 69	265.0 (72.4)	
WHO grade		
Low-grade (1–2), n = 308	293.1 (93.2)	0.406
High-grade (3), n = 46	305.4 (96.3)	
Stage		
Stage I, n = 81	275.0 (69.6)	< 0.001
Stage II, n = 113	296.8 (91.8)	
Stage III, n = 116	283.5 (87.6)	
Stage IV, n = 45	351.2 (122.0)	
T class		
T1, n = 15	271.4 (81.7)	0.003
T2, n = 89	270.6 (64.5)	
T3, n = 225	299.1 (95.0)	
T4, n = 26	355.8 (134.4)	
N class		
N0, n = 201	289.2 (84.8)	0.124
N1, n = 96	291.5 (98.0)	
N2, n = 57	317.4 (109.5)	
M class		
M0, n = 311	286.8 (85.7)	0.001
M1, n = 45	351.2 (122.0)	
Lymphatic invasion		
No, n = 192	287.9 (85.7)	0.162
Yes, n = 160	302.1 (101.4)	
Blood vessel invasion		
No, n = 293	291.4 (89.3)	0.263
Yes, n = 59	308.7 (110.8)	
Mismatch repair enzyme status		
Proficient, n = 315	290.1 (92.4)	0.006
Deficient, n = 40	332.9 (94.9)	
BRAF VE1 immunohistochemistry		
Negative, n = 322	292.7 (93.8)	0.126
Positive, n = 33	318.8 (87.3)	
Modified Glasgow Prognostic Score		
mGPS0, n = 269	283.1 (86.3)	< 0.001
mGPS1, n = 63	335.7 (101.5)	
mGPS2, n = 8	413.2 (148.2)	
Anemia		
No, n = 202	264.5 (73.7)	< 0.001
Yes, n = 154	334.8 (101.4)	
Anemia category		
No anemia, n = 202	264.5 (73.7)	0.005
Microcytic anemia, n = 43	372.8 (113.4)	
Normocytic anemia, n = 109	322.6 (91.5)	
Macrocytic anemia, n = 2	181.0 (58.0)	
Aspirin use		
No, n = 271	297.3 (90.5)	0.396
Yes, n = 85	287.4 (102.3)	

### Platelet count and systemic inflammation

We hypothesized that elevated platelet count would be associated with systemic inflammation in CRC. Supporting the hypothesis, increased platelet count, as a continuous variable, was associated with higher mGPS (p < 0.001; Table 1) and with higher serum C-reactive protein levels (univariable p < 0.001; multivariable adjusted p = 0.003; Additional file 1: Table S3).

**Fig. 1:**
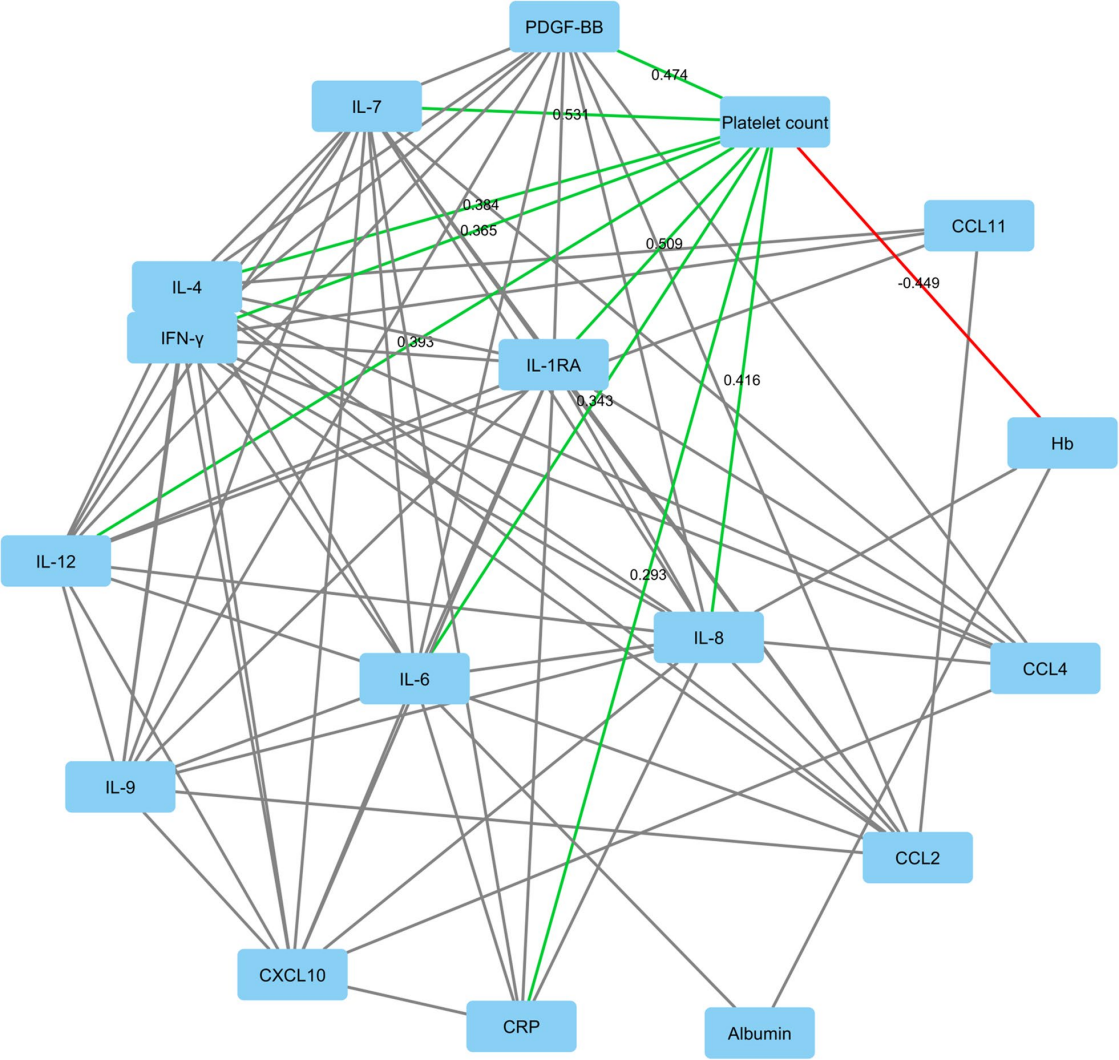
2D visualization of the relationships between blood platelet count, blood hemoglobin levels, serum C-reactive protein levels, serum albumin levels, and serum cytokine levels in a subset of 148 patients. The edges (connecting lines) depict the associations between the variables (only those with p < 0.001 shown). The edge length illustrates the significance of the association. The correlations between platelet count and other variables are represented by green (positive correlation) and red (negative correlation) edges, with the label indicating corresponding Pearson r for the correlation. The other associations are indicated by the grey edges. The 2D visualization was created with Cytoscape software platform [37], utilizing the Prefuse force directed algorithm weighted by the statistical significances of the correlations between individual variables. CCL: chemokine (C–C motif) ligand; CRP: C-reactive protein; CXCL: chemokine (C-X-C motif) ligand; Hb: hemoglobin; IFN: interferon; IL: interleukin; PDGF: platelet-derived growth factor

For more detailed overview on the relationships between platelet count and systemic inflammatory mediators, serum analysis of 13 cytokines was conducted in 148 patients. This analysis indicated that platelet count positively correlated with most of the studied cytokines, including IL-1RA, IL-4, IL-6, IL-7, IL-8, IL-12. IFNγ, and PDGF-BB (univariable and multivariable adjusted p < 0.001 for all; Fig. 1; Table 2); the strongest correlations were with IL-7 (r = 0.531), IL-1RA (r = 0.509), and PDGF-BB (r = 0.474).

**Table 2:** Correlation between blood platelet count and serum cytokine levels

Variable	N (unadjusted, adjusted)	Unadjusted		Adjusted	
		Pearson r	p value	Beta	p value
IL-1RA	148, 146	0.509	< 0.001	0.428	< 0.001
IL-4	148, 146	0.384	< 0.001	0.354	< 0.001
IL-6	147, 145	0.343	< 0.001	0.265	< 0.001
IL-7	148, 146	0.531	< 0.001	0.521	< 0.001
IL-8	148, 146	0.416	< 0.001	0.286	< 0.001
IL-9	147, 145	0.243	0.003	0.237	0.002
IL-12	148, 146	0.393	< 0.001	0.363	< 0.001
IFNγ	148, 146	0.365	< 0.001	0.317	< 0.001
CXCL10	148, 146	0.156	0.058	0.107	0.158
CCL2	147, 145	0.146	0.078	0.150	0.052
CCL4	148, 146	0.159	0.054	0.094	0.213
CCL11	148, 146	0.004	0.959	0.094	0.212
PDGF	148, 146	0.474	< 0.001	0.464	< 0.001

Blood platelet count and serum cytokines were logarithmically transformed because of positive skewness. The adjusted correlations were calculated with multiple linear regression. The correlations were adjusted for tumor location (colon vs. rectum), preoperative radiotherapy or chemoradiotherapy, tumor stage variables (T1–2 vs. T3–4; N0 vs. N1–2; M0 vs. M1), patient age, patient gender, and blood hemoglobin levels

CCL: chemokine (C–C motif) ligand; CXCL: chemokine (C-X-C motif) ligand; IFN: interferon; IL: interleukin; PDGF: platelet-derived growth factor

### Platelet count and tumor infiltrating immune cells

**Fig. 2:**
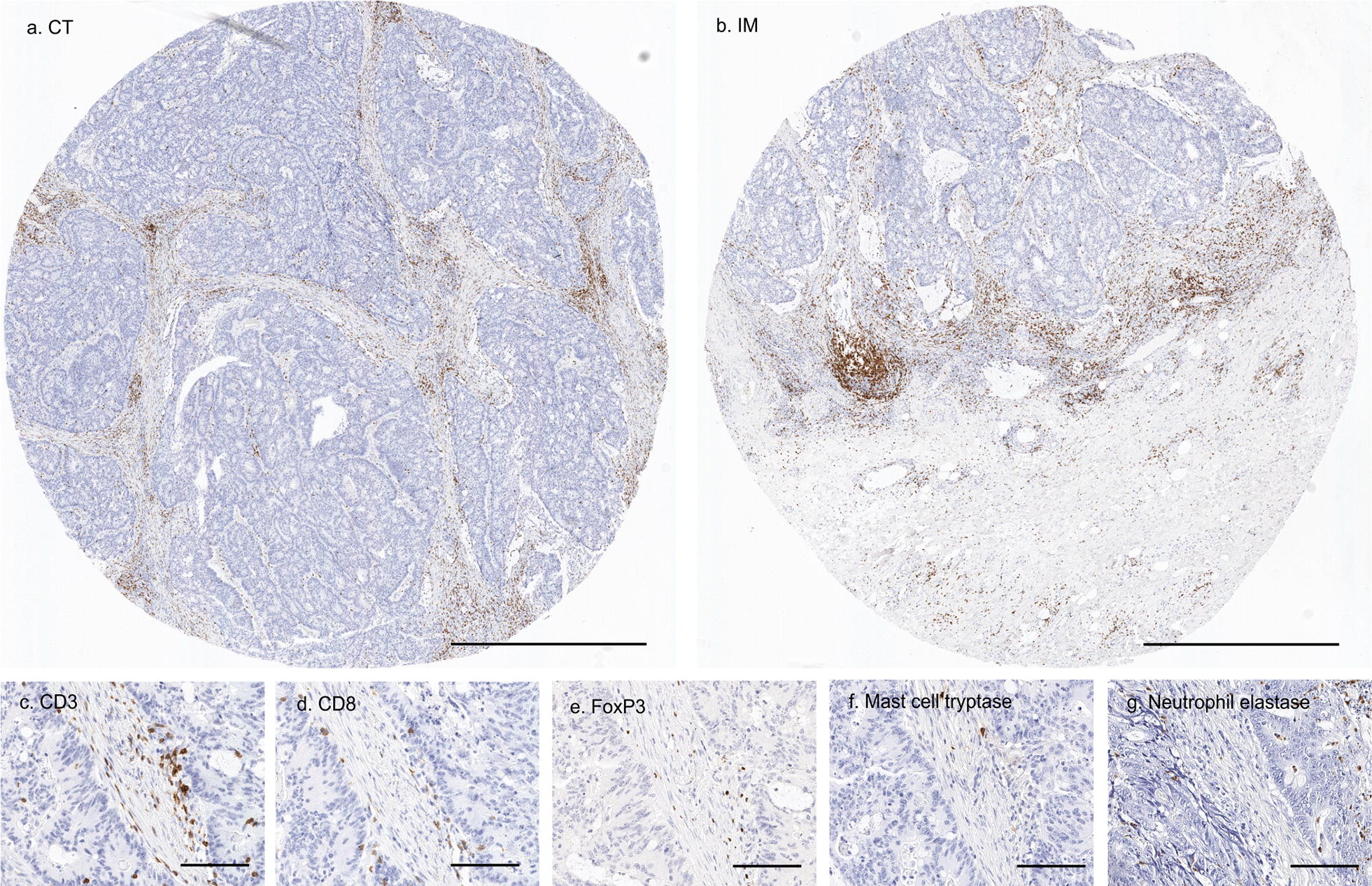
Detection of five types of immune cells in colorectal cancer tissue microarrays with immunohistochemistry. **a**, **b** Examples of tissue microarray cores from the center of the tumor (CT) and the invasive margin (IM) with CD3 immunohistochemistry. Scale bar is 1 mm. **c**–**g** Close-up views portraying CD3^+^ T cells, CD8^+^ cytotoxic T cells, FoxP3^+^ regulatory T cells, Tryptase^+^ mast cells, and Elastase^+^ neutrophils. Scale bar is 100 µm

The strong associations between platelet count and serum cytokine levels lead us to hypothesize that platelet count would also contribute to tumor immune cell infiltration by the release of inflammatory mediators. Thus, correlations between platelet count and five types of tumor infiltrating immune cells (CD3^+^ T cells, CD8^+^ cytotoxic T cells, FoxP3^+^ regulatory T cells, Tryptase^+^ mast cells, Elastase^+^ neutrophils) in different tumor locations were analyzed (Fig. 2; Table 3). However, no statistically significant multivariable adjusted associations were detected between platelet count and the analyzed cell types.

**Table 3:** Correlations between blood platelet count and the densities of tumor infiltrating immune cells

Variable	N (unadjusted, adjusted)	Unadjusted		Adjusted	
		Pearson r	p value	Beta	p value
CD3 IM	354, 352	0.035	0.511	0.033	0.503
CD3 CT	355, 353	− 0.021	0.694	0.015	0.761
CD3 IEL	352, 350	0.058	0.280	0.029	0.565
CD8 IM	355, 353	0.097	0.068	0.057	0.272
CD8 CT	355, 353	− 0.017	0.752	− 0.032	0.513
CD8 IEL	345, 344	0.062	0.247	0.033	0.519
FoxP3 IM	354, 352	− 0.055	0.301	− 0.007	0.899
FoxP3 CT	354, 352	− 0.108	0.043	− 0.054	0.281
Mast cell tryptase IM	354, 352	− 0.119	0.025	− 0.054	0.255
Mast cell tryptase CT	354, 352	− 0.078	0.143	0.008	0.867
Neutrophil elastase IM	347, 345	0.015	0.776	0.035	0.470
Neutrophil elastase CT	347, 345	0.042	0.435	0.000	1.000

Blood platelet count and immune cell densities were logarithmically transformed because of positive skewness. The correlations were adjusted for tumor location (colon vs. rectum), preoperative radiotherapy or chemoradiotherapy, tumor stage variables (T1–2 vs. T3–4; N0 vs. N1–2; M0 vs. M1), patient age, patient gender, and blood hemoglobin levels with multiple linear regression

IM: invasive margin; CT: center of tumor; IEL: intraepithelial

### Aspirin use and colorectal cancer characteristics

We hypothesized that the effects of platelets in CRC could depend on whether patients receive aspirin medication inhibiting platelet activation. Therefore, we analyzed the relationships between aspirin use, clinicopathological characteristics (Additional file 1: Table S4), systemic inflammatory markers (Additional file 1: Table S5), and tumor infiltrating immune cells (Additional file 1: Table S5). There were 85 (23.9%) patients, who had aspirin in their list of daily medications in the clinical records at the time of surgery, with dosage of 100 mg/day (n = 75, 88.2%) or 50 mg/day (n = 10, 11.8%). Aspirin use was more prevalent in the older age group (p < 0.001), among the patients who did not receive preoperative RT/CRT (p = 0.010; borderline statistical significance considering multiple hypothesis testing), in lower N-class (p = 0.026; borderline statistical significance considering multiple hypothesis testing), and in patients with anemia (p = 0.039; borderline statistical significance considering multiple hypothesis testing). Aspirin use did not significantly associate with systemic inflammatory markers (Additional file 1: Table S6) but associated with a tendency towards higher numbers of FoxP3^+^ T cells at the IM (p = 0.005) and higher numbers of neutrophils in the CT (p = 0.031; borderline statistical significance considering multiple hypothesis testing).

### Survival analyses

**Fig. 3:**
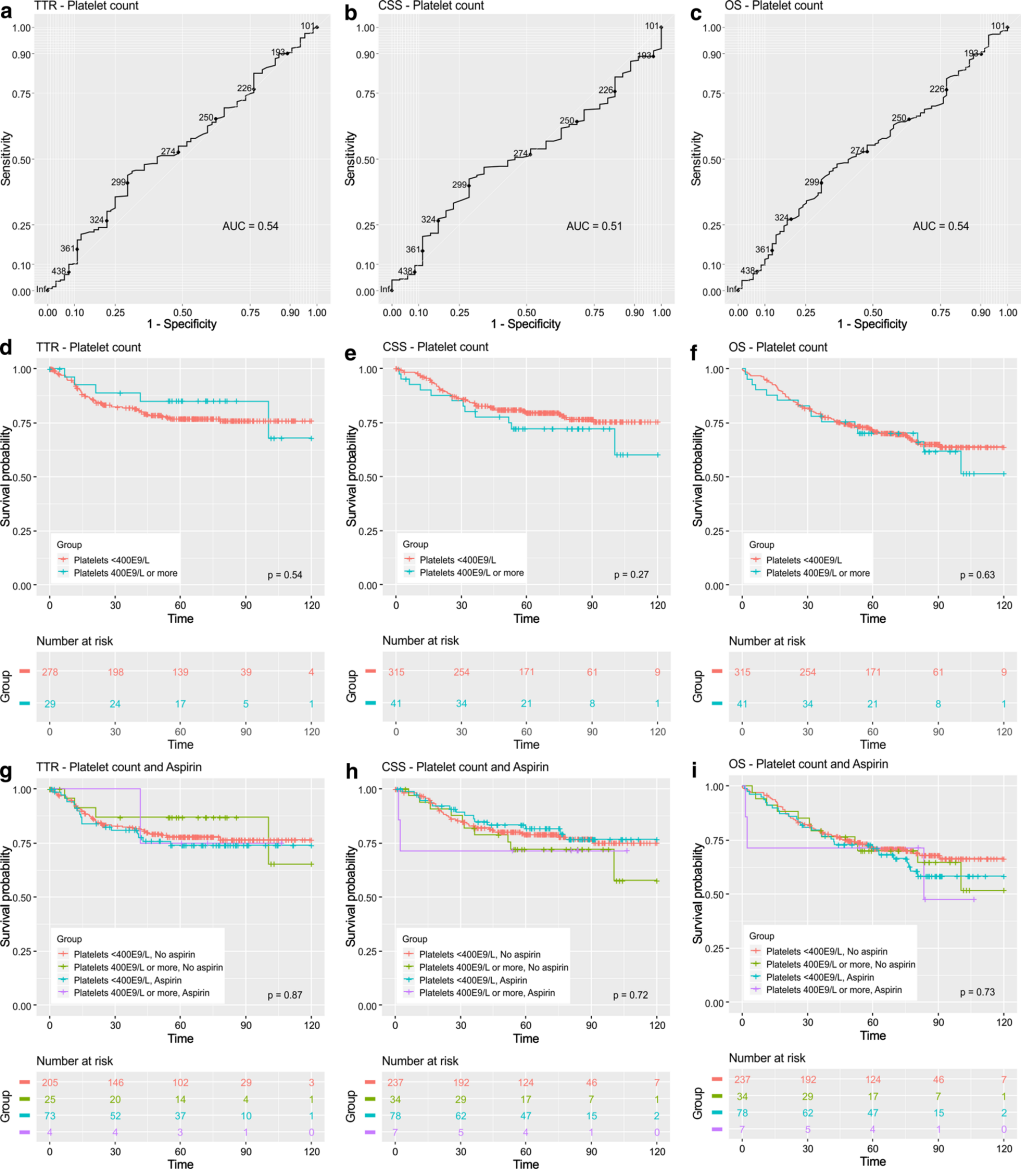
Platelet count, aspirin use, and colorectal cancer survival. **a**–**c** Receiver-operating characteristics (ROC) curves displaying the ability of blood platelet count to distinguish time to recurrence (TTR), cancer specific survival (CSS), and overall survival (OS). **d**–**f** Kaplan–Meier curves showing the relationships between blood platelet count and TTR, CSS, and OS. **g**–**i** Kaplan–Meier curves showing the relationships between combined classification of blood platelet count and aspirin use and TTR, CSS, and OS

Finally, survival analyses were conducted. ROC analysis indicated that blood platelet count did not show good ability to discriminate survivors from non-survivors (Fig. 3a–c). In the previous studies, 400 × 10^9^/L has been the most commonly used cut-off for high platelet count [11], and we decided to apply that to our primary analyses. However, subsequent Kaplan–Meier curves (Fig. 3d–f), and univariable Cox regression models (Additional file 1: Table S6) did not indicate statistically significant association between platelet count and survival. To evaluate the consistency of our data with different cut-off values, we also tested 300 × 10^9^/L and 200 × 10^9^/L as the cut-off points, and the results were parallel to those with 400 × 10^9^/L (Additional file 1: Figs. S2, S3, Table S6). We hypothesized that aspirin use could influence the prognostic effect of platelet count. However, combined evaluation of platelet count and aspirin did not show statistically significant associations with survival (Fig. 3g–i, Additional file 1: Figs. S2, S3, Table S6). Blood platelet count and aspirin use were not statistically significant in multivariable Cox models (Table 4). There was no statistically significant interaction between platelet count and aspirin use (TTR: p for interaction = 0.933, CSS: p for interaction = 0.536, OS: p for interaction = 0.500).

**Table 4:** Cox proportional hazard regression models for time to recurrence (TTR), cancer-specific survival (CSS), and overall survival (OS) according to blood platelet count and clinicopathological characteristics

	TTR^a^			CSS^b^			OS^c^		
	HR	95% CI	p value	HR	95% CI	p value	HR	95% CI	p value
Patient age (< 65 vs. ≥ 65)	1.25	0.72–2.17	0.428	1.90	1.12–3.24	0.018	1.93	1.24–3.00	0.003
Patient gender (male vs. female)	0.93	0.56–1.55	0.794	1.45	0.90–2.32	0.124	1.10	0.76–1.61	0.605
Tumor invasion (T1–T2 vs. T3–T4)	1.86	0.91–3.80	0.091	1.25	0.65–2.39	0.504	1.20	0.74–1.96	0.456
Nodal metastases (N0 vs. N1–N2)	4.89	2.50–9.57	< 0.001	2.66	1.40–5.05	0.003	2.10	1.29–3.39	0.003
Distant metastases (M0 vs. M1)	–	–	–	6.43	3.63–11.4	< 0.001	4.61	2.80–7.62	< 0.001
Tumor location (colon vs. rectum)	1.13	0.61–2.09	0.697	0.86	0.51–1.46	0.577	0.83	0.53–1.31	0.430
Preoperative radiotherapy or chemoradiotherapy (no vs. yes)	1.25	0.61–2.53	0.541	1.43	0.69–2.94	0.334	1.36	0.75–2.46	0.319
Lymphatic invasion (no vs. yes)	1.20	0.64–2.24	0.572	1.89	1.01–3.54	0.046	1.33	0.83–2.14	0.236
mGPS (0 vs. 1–2)	0.69	0.29–1.65	0.402	1.72	1.00–2.96	0.049	1.67	1.07–2.61	0.023
Normocytic anemia (no vs. yes)	0.89	0.47–1.67	0.709	1.34	0.82–2.20	0.246	1.49	1.00–2.22	0.052
Blood platelet count (< 400 × 10^9^/L vs. ≥ 400 × 10^9^/L)	0.77	0.30–1.99	0.594	0.76	0.39–1.48	0.418	0.68	0.38–1.22	0.197
Aspirin (no vs. yes)	1.22	0.68–2.19	0.499	0.94	0.52–1.68	0.833	1.13	0.73–1.75	0.590

The models aimed to enlighten the prognostic value of blood platelet count in CRC, relative to patient age and gender, TNM variables, lymphatic invasion, systemic inflammation (mGPS), normocytic anemia, aspirin use

CI: confidence interval; CSS: cancer specific survival; HR: hazard ratio; mGPS: modified Glasgow Prognostic Score; OS: overall survival; TTR: time to recurrence

^a^n = 306; median follow-up time 60.4 months (IQR 25.0–79.8); 64 (20.9%) events; 50 (14.0%) cases excluded from the analysis because the operation was not radical or no follow-up data available

^b^n = 356; median follow-up time 64.5 months (IQR 37.3–85.6); 77 (21.6%) events

^c^n = 356; median follow-up time 64.5 months (IQR 37.3–85.6); 114 (32.0%) events

## Discussion

Platelets have been implicated with an important role in cancer pathogenesis, especially in regulating inflammatory reactions. The main findings of this study indicate that high platelet count is associated with elevated systemic inflammatory markers in CRC patients. Platelet count, however, was not statistically significantly associated with the density of tumor-infiltrating immune cells or CRC patient survival.

Systemic inflammation can facilitate tumor progression and metastasis [21]. One of the best validated systemic inflammatory prognostic markers, mGPS is comprised of serum CRP and serum albumin [29, 39]. The results of this study indicate that high platelet count is associated with elevated mGPS and serum CRP levels, supporting the role of thrombocytosis in cancer associated systemic inflammation.

For a more detailed analysis of the relationships between platelet count and systemic inflammatory regulators, we measured serum levels of 13 cytokines. We utilized Cytoscape, an open source software platform for visualizing complex networks [37], in creating a 2D visualization of the relationships between blood platelet count and serum cytokine levels, since visual information can facilitate the comprehension of large quantities of correlation data [40]. The analysis showed that there were strong positive correlations between platelet count and multiple serum cytokine levels, most notably with IL-7, IL-1RA, and PDGF-BB. The mechanisms underlying the observed correlations between platelet count and serum cytokines were not addressed in our study. Thus, these correlations may indicate that (a) platelets contribute to the production of these cytokines or store and release these cytokines, (b) these cytokines contribute to the increased platelet production, or (c) some other unrecognized factors such as shared background factors are involved.

Platelet granules are packed with enzymes, growth factors and cytokines, which are released on platelet activation [41]. Accordingly, the concentrations of many cytokines in plasma and serum samples are not equal. For example, among the cytokines showing high correlation with platelet count in this study, IL-7 and PDGF-BB are found in platelet granules [41, 42]. A recent study utilized the same Bio-Rad cytokine panel that was used in this study to compare the cytokine levels in serum and plasma of healthy subjects [43]. The results indicated that the levels of PDGF-BB, IL-4, IL-7, and CXCL10 were significantly higher in serum samples compared to heparin plasma, suggesting that these cytokines are released from platelets during clotting. However, the same study did not show statistically significant difference in serum levels of IL-1RA, IL-6, IL-8, IL-9, IL-12, IFN-γ, CCL2, CCL4, and CCL11, relative to heparin plasma levels. Of the cytokines potentially released from platelet granules, IL-7 is a major regulator of T cell homeostasis, whereas its role in malignancy is controversial [44]. PDGF-BB exerts growth factor functions during embryonal development and adult tissue homeostasis and repair such as wound healing, whereas in cancer, this molecule can directly stimulate the growth and survival of tumor cells and tumor stromal cells [45]. IL-4 is an anti-inflammatory cytokine, which can also contribute to the survival of CRC stem cells [46]. Thus, the particles released by platelets can contribute to the regulation of tumor growth and tumor associated inflammatory reactions.

Thrombopoietin is regarded as a key hormone in megakaryocyte differentiation and proliferation, resulting in platelet production [8]. The process is closely regulated, since thrombopoietin in plasma binds to the circulating platelet surface receptors and only the remaining free thrombopoietin is available to promote megakaryocyte proliferation [8]. Hepatocytes are a major source of thrombopoietin, and inflammation, particularly IL-6, increases thrombopoietin production [8]. Accordingly, we observed positive correlation between platelet count and serum IL-6 levels, potentially reflecting the up-regulation of thrombopoietin production caused by increased availability of IL-6 in CRC. Notably, our earlier study has indicated that IL-6 is among the cytokines showing the highest increase in CRC, as well as the strongest association with high tumor stage [22]. Thus, thrombocytosis is one of the potential systemic consequences of cancer-induced liver-reprogramming. The potential effects of cytokines on platelets are not limited to platelet production, since a recent study indicated that the presence of cytokines such as IL-6 and IL-8, both of which show positive correlation with platelet count in our data, can also alter platelet structure causing platelet hyperactivation [47].

An anti-tumor immune response can control cancer growth [48], and increased density of tumor infiltrating lymphocytes has been associated with improved survival in CRC [18, 19]. We hypothesized that, by the release of soluble inflammatory mediators, platelets could contribute to the regulation of tumor immune cell infiltration. However, no statistically significant multivariable adjusted correlations were observed between platelet count and the densities of tumor infiltrating CD3^+^, CD8^+^, and FoxP3^+^ T cells, neutrophils, or mast cells. This suggests that platelet count only account for a minority of the changes in the densities of tumor infiltrating immune cells, while other factors such as microsatellite instability associated with increased tumor immunogenicity are more important [49, 50].

Improved prognostic parameters are needed to classify CRC into more homogenous and therapeutically relevant groups. Three recent meta-analyses [9–11] suggest that elevated preoperative platelet count is associated with decreased survival in CRC. In our present study, higher platelet counts were observed in higher tumor stage, but platelet count was not significantly associated with survival. Thus, the results do not support the prognostic value of platelet count in unselected stage I–IV CRC patients. Our sample size did not enable comprehensible evaluation of the effect of platelet count in more specific patient subgroups, such as stage II patients. Relative to earlier studies, the major advantage of this study was the inclusion of vast number of additional prognostic parameters such as lymphatic and blood vessel invasion, anemia, and mGPS, for comparison. In addition to elevated systemic inflammatory markers, low blood hemoglobin level was one of the best determinants of high platelet count in this study. The association between reactive thrombocytosis and anemia is also well-known in general population but its pathophysiology remains incompletely understood [51]. We observed high platelet count in patients with either normocytic or microcytic anemia. Microcytic anemia is most commonly due to iron deficiency, while chronic inflammatory conditions are among the most common causes of normocytic anemia in general population [52]. In CRC patients, not only normocytic but also microcytic anemia, is associated with systemic inflammation [23, 53]. Both systemic inflammation and anemia have been associated with adverse clinical outcome in CRC [29, 53–55], and this study highlights the importance to include them as comparison for platelet count also in the subsequent studies evaluating the prognostic significance of platelet count.

Inhibiting cyclooxygenase enzymes and thus platelet activation, aspirin is frequently used in cardiovascular event prevention [56]. In the setting of cancer, aspirin use has been associated with decreased CRC incidence and improved CRC outcome [12]. We hypothesized that the beneficial effect of aspirin in CRC could be limited to patients with high platelet counts. However, we did not observe significantly improved survival in the patients who received aspirin regardless of the platelet count. Small patient numbers in some subgroups, in particular, in the aspirin treated group with high platelet count, might have contributed to this negative result, increasing the risk of type 2 error. Moreover, our sample size did not enable us to analyze, whether different aspirin dosages (100 mg/day or 50 mg/day) were differentially associated with tumor or patient characteristics or survival. Thus, additional studies with larger cohorts are warranted. In addition, the aspirin use data was based on medical records, and potential misclassification due to missing data on over the counter aspirin use has to be taken into account.

Also, some other limitations need to be considered in the interpretation of our results. First, tissue microarrays were used in the immunohistochemical analyses. As only a minor fraction of tumor tissue is analyzed, this may not be representative of the whole tissue section. However, numerous studies have generated reproducible results using tissue microarrays [57]. Moreover, our tissue microarrays were optimized for immune cell counting, including large (diameter 3.0 mm) cores from different parts of the tumor (Fig. 2). An accurate, earlier validated computer assisted immune cell counting method was used [35]. Second, multiple hypotheses were tested in this observational study. However, we adjusted the level of statistical significance of our exploratory analyses to p = 0.005 [38] and interpreted the results with p = 0.05–0.005 (considered borderline statistical significance) cautiously. This approach results in some increase in type 2 statistical error but reduce the risk of type 1 error. The advantages were a prospectively recruited study population, with consistent and extensive histological analysis, including additional prognostic parameters such as lymphatic invasion, and systemic inflammatory markers. An assemblage of tumor infiltrating immune cell types and serum cytokines were analyzed, creating a detailed view on the relationships between platelet count and inflammatory markers.

## Conclusions

In conclusion, blood platelet count is associated with systemic inflammation in CRC but not significantly with the densities of tumor infiltrating T cells. Especially, platelet count and serum cytokine levels are closely related. Our results do not support the prognostic value of platelet count in general stage I–IV material, but further comparative analyses including blood platelet count and Hb levels, as well as systemic inflammatory markers in more specific patient subgroups, such as stage II patients, are warranted. This study could not demonstrate associations between aspirin use and platelet count, systemic inflammation, or CRC molecular characteristics.

## Additional file

**Additional file 1.** Additional Tables S1–S6 and Figures S1–S3.

## Data Availability

The patients recruited to this study have given their consent that does not allow us to make the data publicly available according to current data protection legislation.

## Abbreviations

CCL: chemokine (C–C motif) ligand
CI: confidence interval
CRC: colorectal cancer
CRP: C-reactive protein
CSS: cancer specific survival
CT: center of tumor
CXCL: chemokine (C-X-C motif) ligand
Hb: hemoglobin
HR: hazard ratio
IFN: interferon
IL: interleukin
IM: invasive margin
mGPS: modified Glasgow Prognostic Score
MMR: mismatch repair
OS: overall survival
PDGF: platelet-derived growth factor
ROC: receiver operating characteristics
RT/CRT: radiotherapy or chemoradiotherapy
SD: standard deviation
TNM: tumor, node, metastasis
TTR: time to recurrence
WHO: World Health Organization
