# Supplementary material for: Platelet count, aspirin use, and characteristics of host inflammatory responses in colorectal cancer
Source: J Transl Med. 2019 Jun 13;17:199. doi: 10.1186/s12967-019-1950-z (PMC6567577; doi:10.1186/s12967-019-1950-z)
Supplement: Supplementary file 1 — Additional file 1. Additional Tables S1–S6 and Figures S1–S3. [file 12967_2019_1950_MOESM1_ESM.pdf]

Additional file 1. Additional Tables S1–S6 and Figures S1–S3

Platelet count, aspirin use, and characteristics of host inflammatory responses in colorectal cancer

Juha P. Väyrynen[1,2], Sara A. Väyrynen[1,3], Päivi Sirniö[1], Ilkka Minkkinen[1], Kai Klintrup[4], Toni Karhu[5], Jyrki Mäkelä[4], Karl-Heinz Herzig[5,6], Tuomo J. Karttunen[1], Anne Tuomisto[1], Markus J. Mäkinen[1]

[1] Cancer and Translational Medicine Research Unit, University of Oulu, POB 5000, Oulu 90014, Finland; Finland; Department of Pathology, Oulu University Hospital and Medical Research Center Oulu, Oulu, POB 21, Oulu 90029, Finland
[2] Department of Oncologic Pathology, Dana-Farber Cancer Institute and Harvard Medical School, 450 Brookline Ave, Boston, MA 02215, USA
[3] Department of Medical Oncology, Dana-Farber Cancer Institute and Harvard Medical School, 450 Brookline Ave, Boston, MA 02215, USA
[4] Research Unit of Surgery, Anesthesia and Intensive Care, University of Oulu, POB 5000, Oulu 90014, Finland; Department of Surgery, Oulu University Hospital and Medical Research Center Oulu, POB 21, Oulu 90029, Finland
[5] Research Unit of Biomedicine and Biocenter of Oulu, University of Oulu, POB 5000, Oulu 90014, Finland; Oulu University Hospital and Medical Research Center Oulu, POB 21, Oulu 90029, Finland
[6] Department of Gastroenterology and Metabolism, Poznan University of Medical Sciences, ul. Szpitalna 27/33, 60-572, Poznan, Poland

Table S1. Characteristics of the colorectal cancer patients

	All patients (n=356)	Patients with serum cytokine measurements (n=148)	Patients without serum cytokine measurements (n=208)
**Age**, mean (SD)	68.2 (11.8)	66.7 (11.1)	69.2 (12.2)
**Gender**			
Male	190 (53.4%)	80 (54.1%)	110 (52.9%)
Female	166 (46.6%)	68 (45.9%)	98 (47.1%)
**Tumor location**			
Proximal colon	123 (34.6%)	48 (32.4%)	75 (36.1%)
Distal colon	73 (20.5%)	28 (18.9%)	45 (21.6%)
Rectum	160 (44.9%)	72 (48.6%)	88 (42.3%)
**Preoperative radiotherapy or chemoradiotherapy**			
No	286 (80.3%)	116 (78.4%)	170 (81.7%)
Yes	70 (19.7%)	32 (21.6%)	38 (18.3%)
**WHO grade**			
Grade 1	79 (22.3%)	21 (14.3%)	58 (28%)
Grade 2	229 (64.7%)	108 (73.5%)	121 (58.5%)
Grade 3	46 (13%)	18 (12.2%)	28 (13.5%)
**TNM Stage**			
Stage I	81 (22.8%)	27 (18.4%)	54 (26%)
Stage II	113 (31.8%)	55 (37.4%)	58 (27.9%)
Stage III	116 (32.7%)	45 (30.6%)	71 (34.1%)
Stage IV	45 (12.7%)	20 (13.6%)	25 (12%)
**Blood platelet count,** (10^9/L), mean (SD)	294.9 (93.4)	303.5 (97.1)	288.8 (90.4)
**Aspirin use**			
No	271 (76.1%)	111 (75%)	160 (76.9%)
Yes	85 (23.9%)	37 (25%)	48 (23.1%)

Table S2. Antibodies and protocols used in immunohistochemistry

Cell type	Antigen	Antigen retrieval	Antibody type	Manufacturer	Clone	Code	Dilution	Incubation	Antibody visualization
T cells	CD3	Tris-EDTA pH9*	mouse monoclonal	Novocastra	PS1	NCL-CD3-PS1	1:50	30 min	EnVision
Cytotoxic T cells	CD8	Tris-EDTA pH9*	mouse monoclonal	Novocastra	4B11	NCL-CD8-4B11	1:200	30 min	EnVision
Regulatory T cells	FoxP3	Tris-EDTA pH9*	mouse monoclonal	Abcam	236A/E7	ab20034	1:100	30 min	EnVision
Neutrophils	Neutrophil elastase	Tris-EDTA pH9*	mouse monoclonal	DAKO	NP57	M0752	1:200	30 min	EnVision
Mast cells	Mast cell tryptase	Tris-EDTA pH9*	mouse monoclonal	DAKO	AA1	M7052	1:2000	30 min	EnVision
$MLH1^+$ cells	MLH1	Tris-EDTA pH9*	mouse monoclonal	Novocastra	ES05	NCL-L-MLH1	1:100	90 min	EnVision
$MSH2^+$ cells	MSH2	Tris-EDTA pH9*	mouse monoclonal	BD-Pharmingen	G219-1129	556349	1:200	60 min	EnVision
$MSH6^+$ cells	MSH6	Tris-EDTA pH9*	mouse monoclonal	BD-Pharmingen	44/MSH6	610919	1:150	90min	EnVision
$PMS2^+$ cells	PMS2	Tris-EDTA pH9*	mouse monoclonal	BD-Pharmingen	A16-4	556415	1:100	90 min	EnVision
BRAF V600E mutated cells	BRAF V600E	Ventana CC1	mouse monoclonal	Spring Bioscience	VE1	E19292	1:2000	32 min	Optiview

*In a microwave oven at 800W for 2 min and at 150W for 15 min.

Table S3. Correlation between blood platelet count and systemic inflammatory markers.

Variable	N (unadjusted, adjusted)	Unadjusted		Adjusted	
		Pearson r	p value	Beta	p value
Serum CRP	337, 335	0.266	<0.001	0.149	0.003
Serum Albumin	356, 354	-0.104	0.050	0.019	0.694

Blood platelet count and serum CRP was logarithmically transformed because of positive skewness. The correlations were adjusted for tumor location (colon vs. rectum), preoperative radiotherapy or chemoradiotherapy, tumor stage variables (T1-2 vs. T3-4; N0 vs. N1-2; M0 vs. M1), patient age, patient gender, and blood hemoglobin levels with multiple linear regression. Abbreviations: CRP: C-reactive protein.

Table S4. Relationships between aspirin use and clinicopathological characteristics.

Variable (n)	No aspirin	Aspirin	P value
**Age**			
<65 (n=130)	118 (90.8%)	12 (9.2%)	<0.001
≥65 (n=226)	153 (67.7%)	73 (32.3%)	
**Sex**			
Male (n=190)	142 (74.7%)	48 (25.3%)	0.511
Female (n=166)	129 (77.7%)	37 (22.3%)	
**Body mass index**			
<25 (n=123)	96 (78.0%)	27 (22.0%)	0.332
25-30 (n=146)	112 (76.7%)	34 (23.3%)	
>30 (n=78)	54 (69.2%)	24 (30.8%)	
**Location of tumor**			
Proximal colon (n=123)	91 (74.0%)	32 (26.0%)	0.160
Distal colon (n=73)	51 (69.9%)	22 (30.1%)	
Rectum (n=160)	129 (80.6%)	31 (19.4%)	
**Preoperative radiotherapy or chemoradiotherapy in rectal cancer patients**			
No (n=91)	67 (73.6%)	24 (26.4%)	0.010
Yes (n=69)	62 (89.9%)	7 (10.1%)	
**WHO grade**			
Grade 1 (n=79)	59 (74.7%)	20 (25.3%)	0.871
Grade 2 (n=229)	176 (76.9%)	53 (23.1%)	
Grade 3 (n=46)	34 (73.9%)	12 (26.1%)	
**TNM Stage**			
Stage I (n=81)	62 (76.5%)	19 (23.5%)	0.629
Stage II (n=113)	82 (72.6%)	31 (27.4%)	
Stage III (n=116)	89 (76.7%)	27 (23.3%)	
Stage IV (n=45)	37 (82.2%)	8 (17.8%)	
**Primary tumor**			
T1 (n=15)	10 (66.7%)	5 (33.3%)	0.575
T2 (n=89)	69 (77.5%)	20 (22.5%)	
T3 (n=225)	169 (75.1%)	56 (24.9%)	
T4 (n=26)	22 (84.6%)	4 (15.4%)	
**Lymph node metastasis**			
N0 (n=201)	150 (74.6%)	51 (25.4%)	0.026
N1 (n=96)	68 (70.8%)	28 (29.2%)	
N2 (n=57)	51 (89.5%)	6 (10.5%)	
**Distant Metastasis**			
M0 (n=311)	234 (75.2%)	77 (24.8%)	0.305
M1 (n=45)	37 (82.2%)	8 (17.8%)	
**Lymphatic invasion**			
No (n=192)	148 (77.1%)	44 (22.9%)	0.748
Yes (n=160)	121 (75.6%)	39 (24.4%)	
**Blood vessel invasion**			
No (n=293)	225 (76.8%)	68 (23.2%)	0.715

Yes (n=59)	44 (74.6%)	15 (25.4%)	
**Mismatch repair (MMR) enzyme status**			
MMR Proficient (n=315)	242 (76.8%)	73 (23.2%)	0.341
MMR Deficient (n=40)	28 (70.0%)	12 (30.0%)	
**BRAF VE1 immunohistochemistry**			
Negative (n=322)	248 (77.0%)	74 (23.0%)	0.346
Positive (n=33)	23 (69.7%)	10 (30.3%)	
**Modified Glasgow Prognostic Score (mGPS)**			
0 (n=269)	207 (77.0%)	62 (23.0%)	0.201
1 (n=63)	49 (77.8%)	14 (22.2%)	
2 (n=8)	4 (50.0%)	4 (50.0%)	
**Anemia**			
No (n=202)	162 (80.2%)	40 (19.8%)	0.039
Yes (n=154)	109 (70.8%)	45 (29.2%)	
**Anemia category**			
No anemia (n=202)	162 (80.2%)	40 (19.8%)	0.124
Microcytic anemia (n=43)	32 (74.4%)	11 (25.6%)	
Normocytic anemia (n=109)	75 (68.8%)	34 (31.2%)	
Macrocytic anemia (n=2)	2 (100.0%)	0 (0.0%)	

Table S5. Relationships between aspirin use, systemic inflammatory markers, and the densities of tumor infiltrating immune cells

**Variable**	No aspirin	Aspirin	P value
**Systemic inflammatory markers**			
Serum C-reactive protein (mg/L)	2.6 (0.8-8.0)	3.4 (0.9-8.8)	0.672
Serum albumin (g/L)	43.0 (41.0-45.0)	43.0 (40.0-45.0)	0.784
**Serum cytokines** (pg/mL)			
IL-1RA	63.7 (38.1-95.5)	57.4 (36.9-86.2)	0.348
IL-4	0.9 (0.7-1.1)	0.8 (0.7-0.9)	0.086
IL-6	4.9 (3.7-8.6)	4.1 (3.2-6.8)	0.101
IL-7	6.1 (4.3-7.8)	4.8 (4.0-6.4)	0.089
IL-8	12.3 (9.0-17.1)	11.0 (8.5-12.5)	0.117
IL-9	9.2 (6.6-13.8)	6.5 (4.4-12.7)	0.071
IL-12	30.0 (14.5-41.0)	28.2 (13.3-40.0)	0.645
IFN-γ	31.5 (23.9-43.4)	26.5 (20.1-36.6)	0.131
CXCL10	885.2 (677.7-1202.1)	939.0 (767.0-1240.9)	0.369
CCL2	18.0 (12.7-28.1)	14.7 (9.7-22.6)	0.087
CCL4	65.3 (51.2-85.4)	63.5 (51.0-76.1)	0.484
CCL11	131.2 (91.2-180.4)	125.5 (97.6-174.3)	0.847
PDGF-BB	8536.6 (6040.7-11081.4)	8288.9 (5669.8-11324.0)	0.808
**Tumor infiltrating immune cells** (cells/mm^2)			
CD3 IM	520.5 (306.0-791.4)	609.3 (330.6-890.9)	0.245
CD3 CT	387.9 (205.7-671.2)	422.0 (235.3-787.3)	0.385
CD3 IEL	16.9 (6.1-54.2)	24.3 (7.0-66.7)	0.232
CD8 IM	135.5 (49.0-306.5)	162.2 (93.1-339.8)	0.111
CD8 CT	80.3 (25.2-183.2)	99.7 (34.3-252.5)	0.311
CD8 IEL	11.8 (2.7-42.2)	12.1 (3.5-50.2)	0.425
FoxP3 IM	134.0 (63.3-233.0)	198.3 (111.8-282.9)	0.005
FoxP3 CT	130.5 (62.3-246.8)	133.7 (76.4-274.6)	0.429
Mast cell tryptase IM	45.1 (27.6-74.4)	37.7 (24.4-67.2)	0.261
Mast cell tryptase CT	30.6 (17.6-56.3)	30.8 (14.6-47.8)	0.211
Neutrophil elastase IM	27.2 (7.0-125.6)	46.8 (12.5-224.9)	0.079
Neutrophil elastase CT	18.2 (5.3-73.0)	38.2 (9.7-86.1)	0.031

Numbers indicate median (IQR). Abbreviations: CCL: Chemokine (C-C motif) ligand; CT: center of tumor; CXCL: Chemokine (C-X-C motif) ligand; IEL: intraepithelial. IFN: interferon IL: interleukin; IM: invasive margin; MCV: mean corpuscular volume; PDGF: Platelet-derived growth factor.

Table S6. Univariable analysis of time to recurrence (TTR), cancer-specific survival (CSS), and overall survival (OS) according to blood platelet count with different cut-off points.

	**TTR[A]**			**CSS[B]**			**OS[C]**		
	HR	95% CI	p value	HR	95% CI	p value	HR	95% CI	p value
**Blood platelet count**									
<200×10^9/L vs. ≥200×10^9/L	0.74	0.37-1.50	0.404	1.37	0.59-3.14	0.463	0.97	0.53-1.76	0.919
<300×10^9/L vs. ≥300×10^9/L	0.69	0.40-1.18	0.174	1.15	0.73-1.81	0.540	0.99	0.68-1.45	0.978
<400×10^9/L vs. ≥400×10^9/L	0.75	0.30-1.87	0.540	1.41	0.76-2.61	0.273	1.14	0.66-1.97	0.627
**Aspirin use**									
No vs. Yes	1.19	0.69-2.04	0.541	0.90	0.53-1.55	0.709	1.23	0.81-1.84	0.330
**Blood platelet count (cut-off 300×10^9/L) and aspirin use**									
Platelet count <300×10^9/L, no aspirin (n=155)	1	reference		1	reference		1	reference	
Platelet count <300×10^9/L, aspirin (n=57)	0.88	0.45-1.70	0.697	0.59	0.27-1.27	0.174	1.07	0.64-1.80	0.797
Platelet count ≥300×10^9/L, no aspirin (n=116)	0.55	0.29-1.05	0.069	0.91	0.54-1.53	0.723	0.92	0.59-1.43	0.700
Platelet count ≥300×10^9/L, aspirin (n=28)	1.20	0.50-2.86	0.681	1.51	0.73-3.13	0.270	1.43	0.76-2.69	0.265
**Blood platelet count (cut-off 400×10^9/L) and aspirin use**									
Platelet count <400×10^9/L, no aspirin (n=237)	1	reference		1	reference		1	reference	
Platelet count <400×10^9/L, aspirin (n=78)	1.16	0.66-2.04	0.604	0.90	0.51-1.60	0.721	1.23	0.80-1.89	0.354
Platelet count ≥400×10^9/L, no aspirin (n=34)	0.74	0.27-2.07	0.569	1.36	0.69-2.67	0.380	1.15	0.62-2.12	0.652
Platelet count ≥400×10^9/L, aspirin (n=7)	1.00	0.14-7.29	0.998	1.49	0.36-6.12	0.582	1.52	0.48-4.84	0.477

[A] n=306; median follow-up time 60.4 months (IQR 25.0-79.8); 64 (20.9%) events; 50 (14.0%) cases excluded from the analysis because the operation was not radical or no follow-up data available.

[B]n=356; median follow-up time 64.5 months (IQR 37.3-85.6); 77 (21.6%) events; 0 (0.0%) cases excluded from the analysis because no follow-up data available.

[C]n=356; median follow-up time 64.5 months (IQR 37.3-85.6); 114 (32.0%) events; 0 (0.0%) cases excluded from the analysis because no follow-up data available.

Abbreviations: CI: confidence interval; CSS: cancer specific survival; HR: hazard ratio; OS: overall survival; TTR: time to recurrence.

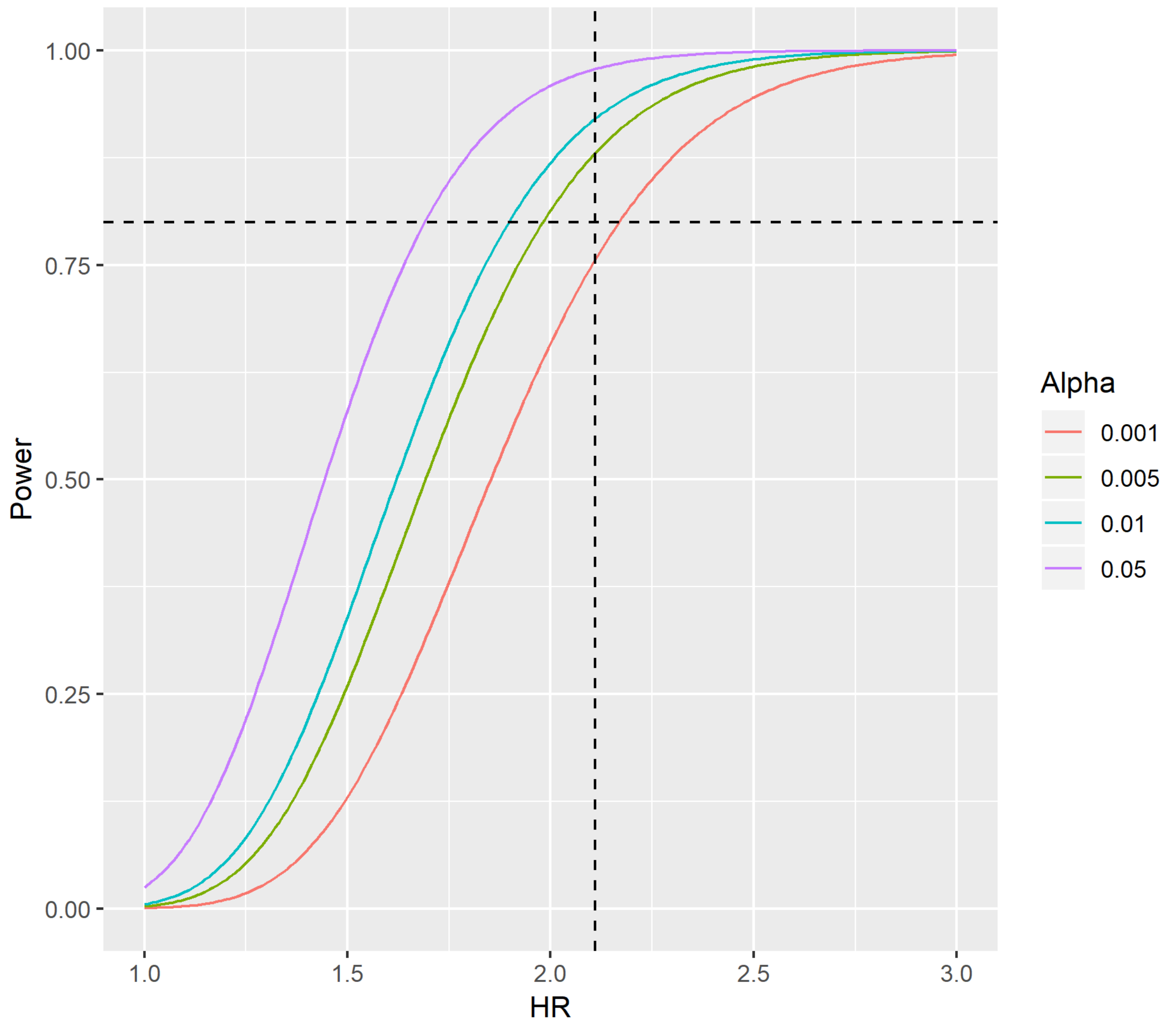

Fig. S1. Estimation of statistical power with different expected hazard ratios (HR) and α levels and in overall survival analysis. Based on the model of Schoenfeld.[1] Assumptions: sample size 356, proportion of patients in the high-risk group 0.5, events in 114 (32.0%) of patients. Potential competing interests were not considered. Horizontal line represents power level of 0.80 (β level 0.20) and vertical line represents expected HR of 2.11, based on a recent meta-analysis.[2]

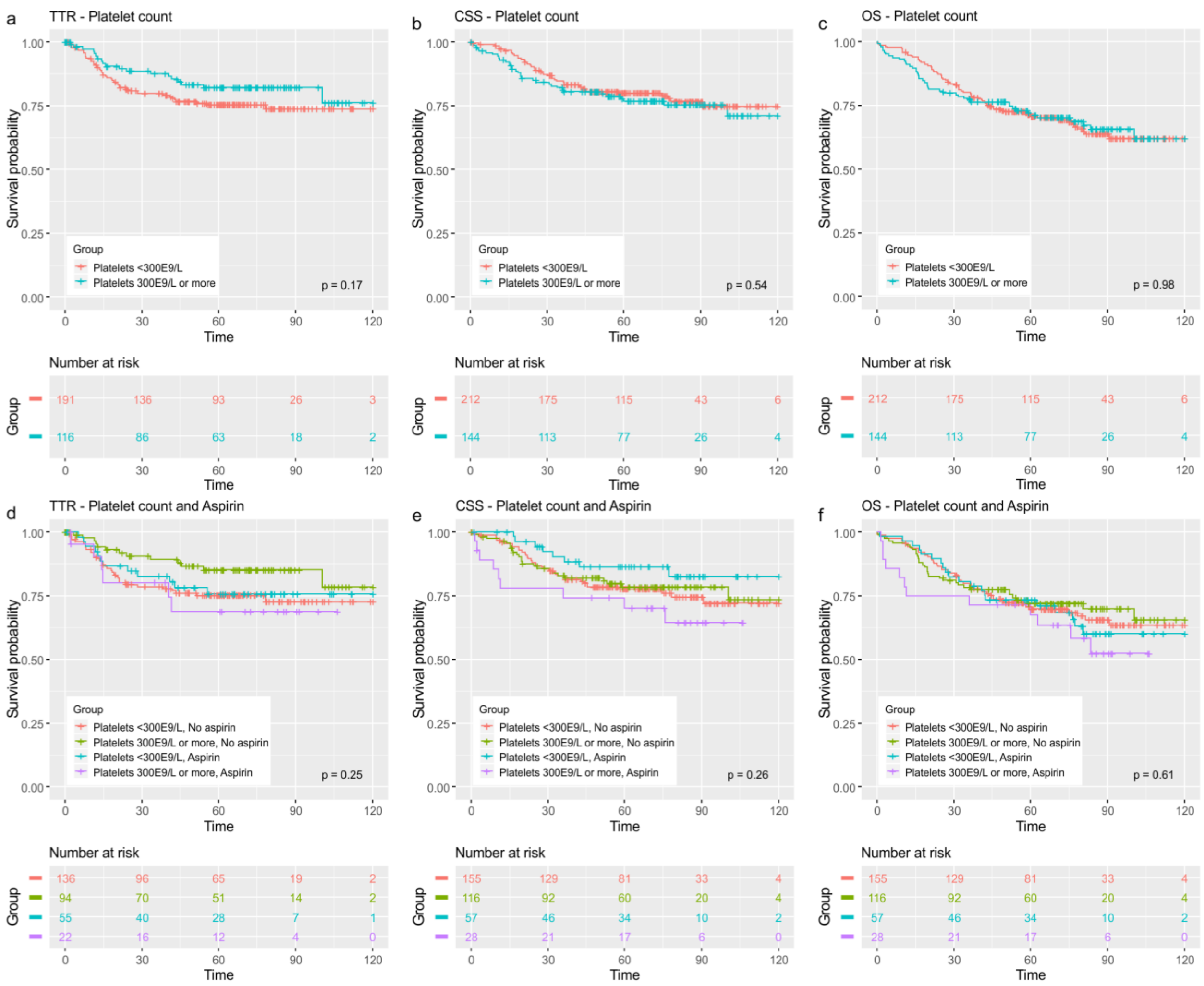

Fig. S2. Platelet count, aspirin use, and colorectal cancer survival with 300×10^{9}/L as the cut-off value for platelet count. a-c. Kaplan-Meier curves showing the relationships between blood platelet count and time to recurrence (TTR), cancer specific survival (CSS), and overall survival (OS). d-f. Kaplan-Meier curves showing the relationships between combined classification of blood platelet count and aspirin use and TTR, CSS, and OS.

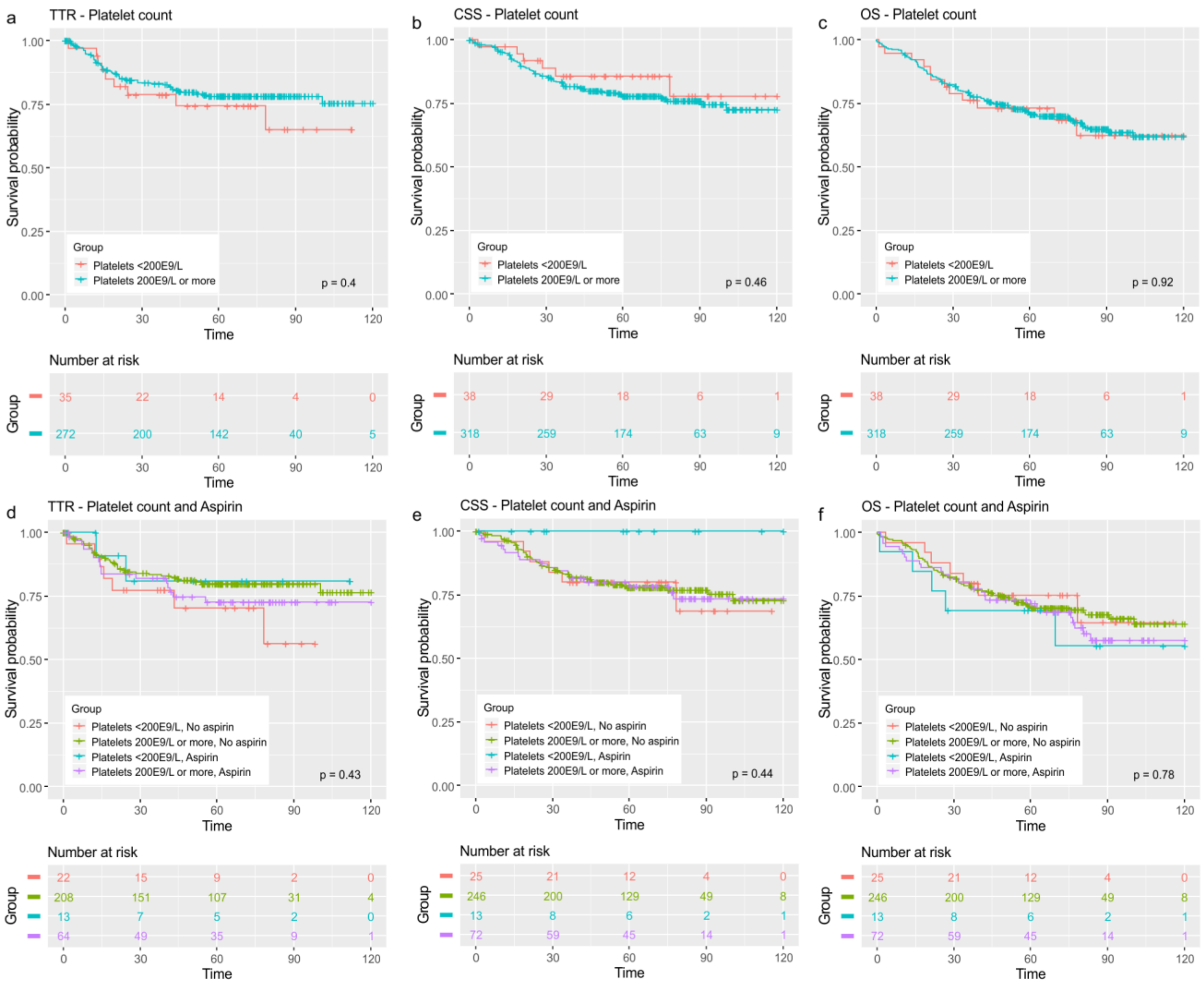

Fig. S3. Platelet count, aspirin use, and colorectal cancer survival with 200×10^9/L as the cut-off value for platelet count. a-c. Kaplan-Meier curves showing the relationships between blood platelet count and time to recurrence (TTR), cancer specific survival (CSS), and overall survival (OS). d-f. Kaplan-Meier curves showing the relationships between combined classification of blood platelet count and aspirin use and TTR, CSS, and OS.

References

1. Schoenfeld DA. Sample-size formula for the proportional-hazards regression model. *Biometrics* 1983;39:499–503.

2. Rao X-D, Zhang H, Xu Z-S, Cheng H, Shen W, Wang X-P. Poor prognostic role of the pretreatment platelet counts in colorectal cancer: A meta-analysis. *Medicine (Baltimore)* 2018;97:e10831.
